# Zero‐Dimensional Interstitial Electron‐Induced Spin–Orbit Coupling Dirac States in Sandwich Electride

**DOI:** 10.1002/smsc.202400131

**Published:** 2024-07-05

**Authors:** Weizhen Meng, Jiayu Jiang, Yalong Jiao, Fengxian Ma, Ying Yang, Zhenxiang Cheng, Xiaotian Wang

**Affiliations:** ^1^ College of Physics Hebei Key Laboratory of Photophysics Research and Application Hebei Normal University Shijiazhuang 050024 China; ^2^ State Key Laboratory of Reliability and Intelligence of Electrical Equipment, and School of Materials Science and Engineering Hebei University of Technology Tianjin 300130 China; ^3^ College of Physics and Electronic Engineering Chongqing Normal University Chongqing 401331 China; ^4^ Institute for Superconducting and Electronic Materials (ISEM) Faculty of Engineering and Information Sciences University of Wollongong Wollongong New South Wales 2500 Australia

**Keywords:** Dirac fermions, low‐work function, N_2_ cleavage, 2D electrides

## Abstract

The development of inorganic electrides offers new possibilities for studying topological states due to the nonnuclear‐binding properties displayed by interstitial electrons. Herein, a sandwich electride 2[CaCl]^+^:2e^−^ is designed, featuring a tetragonal lattice structure, including two atomic lattice layers and one interstitial electron layer. The interstitial electrons form nonsymmorphic‐symmetry‐protected Dirac points (DPs) at the X and M points, which are robust against the spin–orbit coupling effect. DPs exhibit an approximately elliptical shape, characterized by a relatively high anisotropy, resulting from the interplay between the electron and atomic layers. In addition, 2[CaCl]^+^:2e^−^ possesses a lower work function (WF) (3.43 eV), endowing it with robust electron‐supplying characteristics. Due to the low WF and interstitial electrons, 2[CaCl]^+^:2e^−^ loaded Ru shows outstanding catalytic performance for N_2_ cleavage. A potential research platform for exploring the formation of topological states and promoting nitrogen cracking in electrides is provided.

## Graphical abstract

The interstitial anionic electrons (IAEs) of 0D inorganic electride 2[CaCl]^+^:2e^−^ form robust spin–orbit coupling Dirac points near Fermi level. Due to the presence of IAEs, 2[CaCl]^+^:2e^−^ exhibits a lower work function, which enhances the dissociation of CaCl@Ru for N_2_.

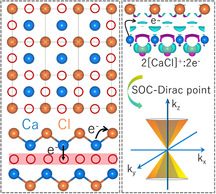

## 1 Introduction

**Figure 1:**
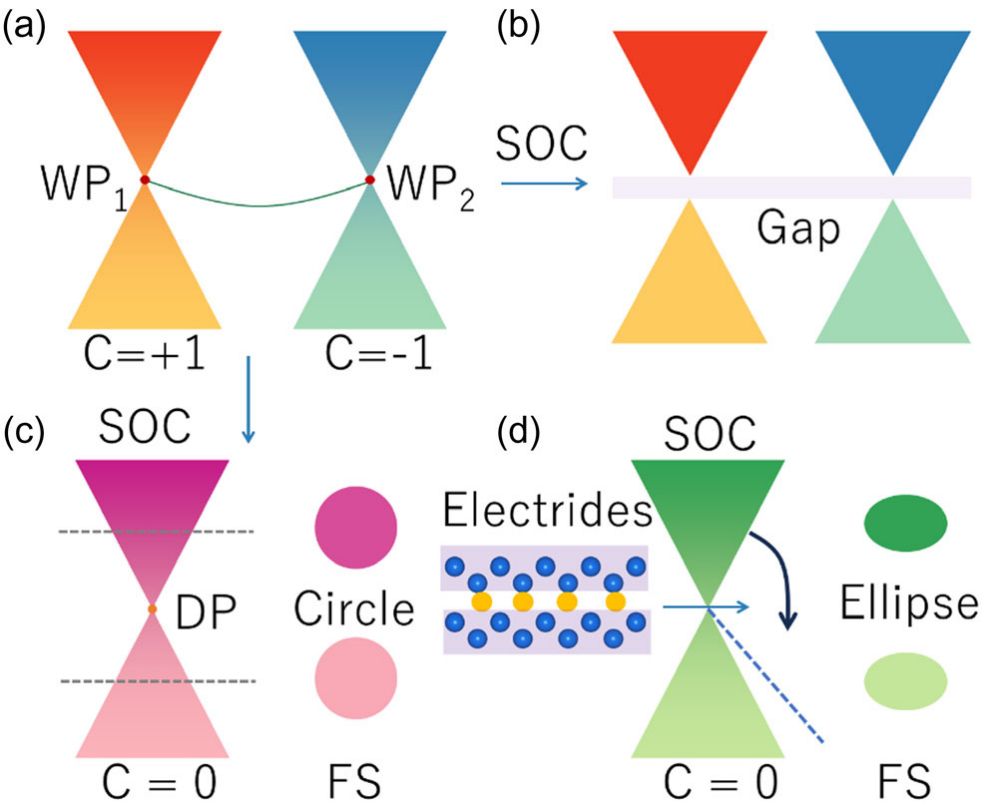
When considering spin–orbit coupling (SOC), a) two Weyl points (WPs) with opposite chirality can form massive DPs b) and also can form massless DPs c). d) Interstitial electrons in inorganic electrides can induce quadratic dispersion in one direction of linear DP, resulting in an elliptic Fermi surface (FS).

Since the discovery of graphene,^[^ ^1^ ^]^ a series of interesting physical properties have been associated with its massless Dirac point (DP) on the Fermi level, such as quantum spin hall effect^[^ ^2^ ^]^ and high Fermi speed.^[^ ^3^ ^]^ Subsequently, researchers began to focus on exploring 2D materials with DPs in the low‐energy region and predicted hundreds of candidates,^[^ ^4^ ^]^ such as silience,^[^ ^5^ ^]^ graphyene,^[^ ^6^ ^]^ FeB_2_,^[^ ^7^ ^]^ and hr‐Sb.^[^ ^8^ ^]^ However, these so‐called 2D Dirac materials are not robust under spin–orbit coupling (SOC). Specifically, the DPs observed in these 2D systems are actually Weyl points (WPs) formed by two single bands with a one‐degree irreducible representation, as shown in **Figure** **1**a. Once considering SOC, the WPs will open different gaps and form a massive DP, as shown in Figure 1b. *Is it possible to possess* 2*D DPs that are robust against SOC?* Young and Kane^[^ ^9^ ^]^ give a positive response that SOC‐DPs can exist in layer groups with nonsymmorphic symmetries.

Motivated by Kane's work, we found that 27‐layer groups can host SOC‐DPs by group theory analysis.^[^ ^10^ ^]^ Besides, these layer groups with SOC‐DPs can be classified into centrosymmetric and non‐centrosymmetric groups. For the centrosymmetric class, DPs [Chern number (C = 0)] are constructed from two WPs with opposite chirality (C = ±1), as shown in Figure 1c. Candidate materials include CuCN_2_,^[^ ^11^ ^]^ X_3_SiTe_6_ (X = Ta, Nb),^[^ ^12^ ^]^ HfGeTe,^[^ ^13^ ^]^ TaCoTe_2_,^[^ ^14^ ^]^ etc. For the non‐centrosymmetric class, DPs with nonzero Berry curvature are formed by four single bands.^[^ ^15^ ^]^ It is predicted in penta‐CN_2_,^[^ ^10^ ^]^ SbSSn,^[^ ^15^ ^]^ etc. Currently, most SOC DPs exhibit linear dispersion, which means forming isotropic Fermi surface (FS) in the low‐energy region, as shown in Figure 1c. However, when linear dispersion occurs in one direction and approximate quadratic dispersion occurs in the other direction, the FS exhibits approximately elliptical morphology (namely high anisotropy), as shown in Figure 1d. Remarkably, compared with linear DPs, DPs with the anisotropic FS can induce unique physical properties, such as anisotropic electron conduction^[^ ^16^ ^]^ and nonlinear anomalous effects.^[^ ^17^ ^]^

In our recent work,^[^ ^18^ ^]^ we found that electrides with a sandwich structure formed by two layers of atomic lattices and one layer of electron sublattices are ideal successors. Inorganic electrides are a special type of electron‐rich materials, excess electrons highly positioned in the interstitial positions formed by cations, acting as anions or interstitial quasi‐atoms.^[^ ^19^, ^20^ ^]^ Due to the approximate nuclear‐free binding of interstitial electrons, inorganic electrides exhibit interesting physical and chemical properties,^[^ ^21^, ^22^, ^23^, ^24^, ^25^ ^]^ such as abundant topological states,^[^ ^26^, ^27^, ^28^ ^]^ unique magnetism,^[^ ^29^, ^30^, ^31^ ^]^ lower work functions,^[^ ^32^, ^33^, ^34^, ^35^, ^36^, ^37^, ^38^ ^]^ higher electron mobility,^[^ ^39^ ^]^ and higher surface electron activity.^[^ ^37^, ^38^, ^40^ ^]^ One can find that compared to traditional functional materials, inorganic electrides exhibit rich characteristics. In addition, a large number of SOC‐DPs are mainly proposed in 2D electrically neutral materials. If SOC‐DPs are found in 2D inorganic electrides, it is of great significance for the applications of topological electrides.

In this work, we designed an inorganic electride with a sandwich structure composed of two atomic lattices and one electron sublattices, namely 2[CaCl]^2+^:2e^−^. Under the constraint of atomic lattice, interstitial electrons form a natural tetragonal lattice. Furthermore, interstitial electrons form two nonsymmorphic‐symmetry‐protected DPs with a four‐degree irreducible representation on X and M points. Due to the presence of interstitial electrons, DPs exhibit an elliptic FS and a lower WF (Φ_WF_  = 3.43 eV). In addition, the strong electron‐donating properties of electrides greatly promote nitrogen cracking by loading Ru metal (overcome the barrier of *N_2_ → *N + *N is 1.59 eV).

## 2 Computational Methods

The numerical calculations in this work were performed within the Vienna ab initio Simulation Package^[^ ^41^ ^]^ in the framework of density‐functional theory (DFT).^[^ ^42^ ^]^ The generalized gradient approximation of the Perdew–Burke–Ernzerhof (PBE) method^[^ ^43^ ^]^ was applied for the exchange‐correlation potential. To avoid interlayer interaction, we build a vacuum space greater than 34 Å for electride 2[CaCl]^2+^:2e^−^. The cutoff energy was adopted as 450 eV, and the BZ was sampled with Γ‐centered k‐point mesh of 11 × 11 × 1. The DFT‐D2 method^[^ ^44^ ^]^ is used to consider the long‐range van der Waals interactions. The PHONOPY code^[^ ^45^ ^]^ was used to calculate the phonon spectra, with a 5 × 5 × 1 supercell and a 5 × 5 × 1 q‐grid. The Fermi surface is calculated by using Wannier functions^[^ ^46^, ^47^ ^]^ the iterative green's function method^[^ ^48^ ^]^ as implemented in the WannierTools package.^[^ ^49^ ^]^

## 3 Results and Discussion

### 3.1 Crystal Structure and Origin of Interstitial Electrons in 2[CaCl]^+^:2e^−^

Figure 1a shows the crystal structure of CaCl. It belongs to the 129‐space group (P4/nmm) with a square structure, corresponding to the 64‐layer group. One can find that a primitive cell contains two Ca atoms and two Cl atoms, occupying Wyckoff sites of (0, 0, 0.40), (0.50, 0.50, 0.52), (0, 0, 0.49), and (0.5, 0.5, 0.36), respectively. Remarkably, a tetrahedral cavity (namely 0D cage) constrained by cationic Ca is formed at the (0, 0, *z*) site (0.4 Å < *z* < 0.49 Å), which is located in the interlayer between two atomic lattices, as shown in Figure 1a. Based on valence state analysis, the valence values of cationic Ca and anionic Cl are +2 (Ca^2+^) and −1 (Cl^−^), respectively. Therefore, CaCl belongs to a typical electron‐rich material, where one primitive cell can accommodate two excess electrons, following the form of 2[CaCl]^+^:2e^−^.

Subsequently, the ELF, CDD, PCD, and DOS were used as judgment methods to verify that CaCl belongs to 0D inorganic electride. As shown in Figure 1b, the ELF of 2[CaCl]^+^:2e^−^ indicates that lattice cavity (0D cage) exists highly localized 0D spherical electrons, thus forming a tetragonal electron sublattice. The CDD indicates that the orbital electrons of cation Ca^2+^ transfer to two destinations, one to the anion Cl^−^, and the other to the interlayer cavity (cage), as shown in Figure 1c. Another feature of electrides is that the bands near the Fermi level are mainly contributed by interstitial electrons. One can clearly observe that the DOS of 2[CaCl]^+^:2e^−^ has a significant contribution of interstitial electrons in the energy range of −2 ≈ 3.8 eV, as shown in Figure 1d. Furthermore, based on PCD analysis, the I‐band is entirely contributed by interstitial electrons, while the II‐band is contributed by both interstitial electrons and a small number of orbital electrons, which agrees well with the results of DOS, as shown in Figure 1e. Based on the above statements, we can firmly determine that 2[CaCl]^+^:2e^−^ is a typical 0D inorganic electride. Before discussing the electronic structure, the phonon spectrum of 2[CaCl]^+^:2e^−^ was calculated, and it was found that there were no virtual frequencies along all *k*‐paths (Γ‐X‐*M*‐Γ), indicating that 2[CaCl]^+^:2e^−^ exhibits high dynamical stability.

### 3.2 Electronic Band Structures of 2[CaCl]^+^:2e^−^

**Figure 2:**
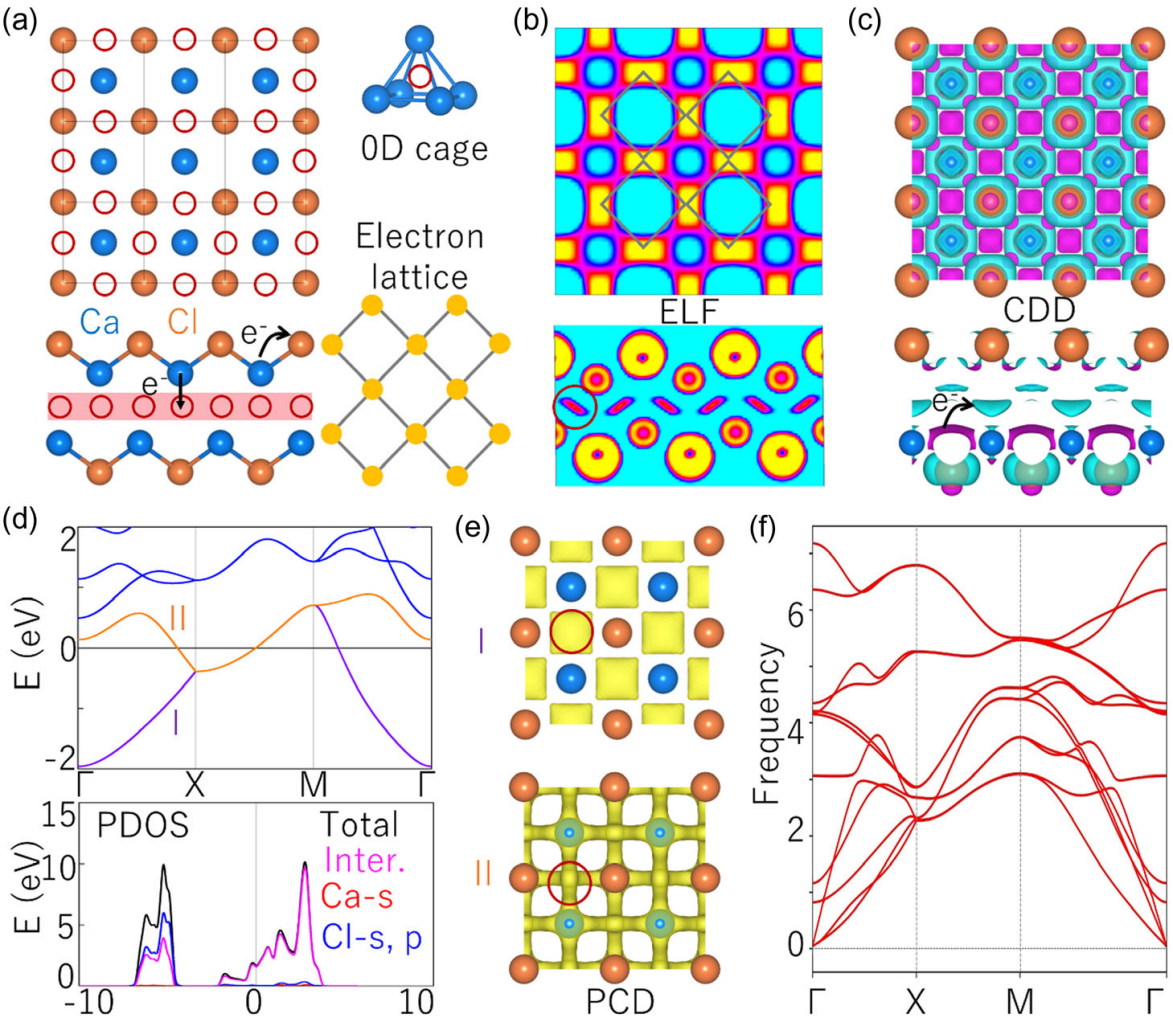
a) Left panel: Side and top views of the crystal structure in 2[CaCl]^+^:2e^−^. Right panel: The orbital electrons of cation Ca^2+^ transfer to the tetrahedron formed by itself, forming a tetragonal electron lattice. b) Electronic localization function (ELF) of 2[CaCl]^+^:2e^−^ with the isosurface values set as 0.75. c) Charge difference density (CDD) of 2[CaCl]^+^:2e^−^, where purple and blue charges represent electron acquisition and electron loss, respectively. d) Electronic band structure and density of states (DOS) of 2[CaCl]^+^:2e^−^, where “inter.” represents “interstitial electrons”. e) Partial charge density (PCD) of I and II‐bands in 2[CaCl]^+^:2e^−^. f) Phonon spectrum of 2[CaCl]^+^:2e^−^.

**Figure 3:**
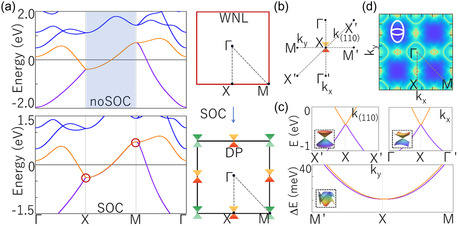
a) Top panel: Electronic band structure of 2[CaCl]^+^:2e^−^ without considering SOC. Bottom panel: Electronic band structure of 2[CaCl]^+^:2e^−^ with SOC. Right panel: Positions of Weyl nodal line (WNL) and DP in Brillouin zone (BZ). c) Electronic band structures along the three directions [*k*_(110)_ (X‐X′), *k*_*x*_ (Γ‐X), and *k*_*y*_ (X‐M)] in (b). d) Fermi surface of 2[CaCl]^+^:2e^−^.

Let us turn our attention to the electronic band structures of 2[CaCl]^+^:2e^−^. Figure 3a shows the electronic band structures of 2[CaCl]^+^:2e^−^ without and with considering spin–orbit coupling (SOC), respectively. One can find that in the absence of SOC, the I and II bands originating from interstitial electrons (**Figure** **2**e) form a double degeneracy along the X‐M path, namely Weyl nodal line (WNL), which is protected by *M*_*z*_: {*x*, *y*, *z*} → {*x* + 1/2, *y* + 1/2, −*z*} (top panel of **Figure** **3**a).

Once considering SOC, the WNL will be split. However, the presence of nonsymmorphic symmetries results in a DP with a four‐degree irreducible representation (*A*_1g_  + *A*_2g_  + *A*_1u_  + *A*_2u_) that is formed at the X and M points, respectively (Bottom panel of Figure 3a). Remarkably, the interstitial electrons of the electride 2[CaCl]^+^:2e^−^ possess an approximate *s*‐orbital property,^[^ ^50^ ^]^ resulting in a very weak splitting of WNL along the X‐M path, as shown in Figure S1, Supporting Information. Subsequently, we calculated the electronic band structure under SOC along *k*_(110)_ (X‐X′), *k*_*x*_ (Γ‐X) and *k*_*y*_ (X‐M) directions, respectively, as shown in Figure 3b,c. One can find that the bands along the *k*_(110)_ and *k*_*x*_ directions show highly linear dispersion, while the bands along the *k*_*y*_ direction show quadratic dispersion in a smaller energy range. This result directly leads to the Fermi surface with an approximate elliptical shape, as shown in Figure 3d. This Fermi surface indicates that DP exhibits extremely high anisotropy and determines a significant difference in Fermi velocity between the linear and quadratic dispersion directions. We have observed similar phenomena in 2[CaCl]^+^:2e^−^ with hexagonal lattice.^[^ ^18^ ^]^ In Section 3.3, we further verify the results of our DFT calculation through symmetry analysis.

### 3.3 Symmetry Analysis

Layer group (LG) 64 can generate the following symmetric operations: ${\tilde{C}}_{2x}:\left\{x,\;y,\;z\right\}\to \left\{x+\frac{1}{2},\;-y,\;-z\right\}$, ${\tilde{C}}_{2y}:\left\{x,\;y,\;z\right\}\to \left\{-x,\;y+\frac{1}{2},\;-z\right\}$, ${\tilde{C}}_{2z}:\left\{x,\;y,\;z\right\}\to \left\{-x+\frac{1}{2},\;y+\frac{1}{2},\;z\right\}$, ${\tilde{C}}_{4z}:\left\{x,\;y,\;z\right\}\to \left\{-y+\frac{1}{2},\;x,\;z\right\}$, ${\tilde{M}}_{y}:\left\{x,\;y,\;z\right\}\to \left\{x+,\;-y+\frac{1}{2},\;z\right\}$, center‐ and time‐inversion symmetries (*PT*). Such that one has an antiunitary relationship along X‐M, namely, ${\left(PT\right)}^{2}=-1$. Furthermore, one also can generate: $\left({\tilde{C}}_{2y}P\right)=e^{-ik_{y}}P{\tilde{C}}_{2y}$. The commutation relationship between any of the two generating elements is

$${\left({\tilde{C}}_{4z}\right)}^{4}=-1,\;{\tilde{C}}_{2y}^{2}=e^{-ik_{y}},\;{\tilde{C}}_{2z}^{2}=-1,\;{\tilde{C}}_{4z}{\tilde{C}}_{2y}=-T_{100}{\tilde{C}}_{2y}{\tilde{C}}_{4z}^{3},\;P{\tilde{C}}_{4z}=T_{100}{\tilde{C}}_{4z}P$$

(1) $${\tilde{C}}_{2y}P=T_{010}P{\tilde{C}}_{2y},\;{\tilde{C}}_{2z}P=T_{110}P{\tilde{C}}_{2z},\;{\tilde{C}}_{2y}{\tilde{C}}_{2z}=-T_{110}{\tilde{C}}_{2z}{\tilde{C}}_{2y}$$

Such that at points X and M can lead to $\left\{{\tilde{C}}_{2y},P\right\}=0$, thus leading to the fourfold degeneracy at X and M points. At X point, we choose the basis as the eigenstates of ${\tilde{C}}_{2z}$ with $c_{2z}=\pm i$, such that,

(2) $${\tilde{C}}_{2z}=i\Gamma_{33},\;P=\Gamma_{10},{\tilde{C}}_{2y}=\Gamma_{30},\;T=i{\;\Gamma }_{02}K$$

Thus, these symmetry operations induce a stable DP, which can be described as

(3) $$H\left(q\right)=-cq_{x}\Gamma_{20}+q_{y}\left(c_{2}\Gamma_{31}-c_{2}\Gamma_{32}\right)$$

At M point, one can generate:

(4) $${\tilde{C}}_{4z}=\frac{1}{\sqrt{2}}\left(\Gamma_{30}+i\Gamma_{33}\right),\;P=\Gamma_{10},{\tilde{C}}_{2y}=\Gamma_{21},\;T=-i{\;\Gamma }_{02}K$$

Thus, these symmetry operations result in a DP given by

(5) $$H\left(q\right)=\left[\begin{array}{cc} \mathrm{q\sigma} & 0\\ 0 & -\mathrm{q\sigma} \end{array}\right]$$

Therefore, the results of symmetry analysis agree well with the results of DFT calculations.

### 3.4 Work Function and Nitrogen Cleavage of 2[CaCl]^+^:2e^−^

**Figure 4:**
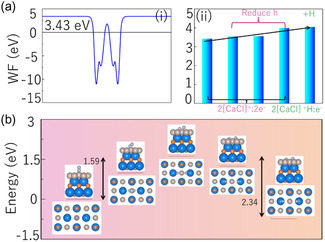
a): (i) Work function (WF) of electride 2[CaCl]^+^:2e^−^. (ii) Changing the WF of 2[CaCl]^+^:2e^−^ by reducing interlayer spacing (h) and introducing paramagnetic atom H. b) Energy profiles for N_2_ activation on Ru/2[CaCl]^+^:2e^−^.

For electrides or unconventional materials,^[^ ^51^, ^52^, ^53^ ^]^ compared to the orbital electrons of atoms, excess electrons are difficult to stabilize under the electric field of atomic nuclei.^[^ ^27^ ^]^ Therefore, inorganic electrides generally exhibit lower WF. In addition, the size effect of quantum can also affect the WF of the material surface, and monolayer materials are usually lower than bulk materials.^[^ ^21^ ^]^ As shown in (i) of **Figure** **4**a, one can find that the WF of 2[CaCl]^+^:2e^−^ is 3.43 eV, which is lower than that of some traditional transition metals and close to or slightly higher than that of some familiar inorganic electrides, as shown in **Table** **1**. Furthermore, inorganic electrides are often utilized to activate certain molecules, such as CO_2_ and N_2_, due to their low WF feature. Subsequently, we confirmed that the lower WF of 2[CaCl]^+^:2e^−^ originated from interstitial electrons by compressing the interlayer spacing (h) and introducing paramagnetic H atoms. As shown in (i) of Figure 4a and Figure S2, Supporting Information, one can find that as the interstitial electrons are gradually annihilated, the WF gradually increases (WF_(2[CaCl]+:2e−)_: 3.43 eV → WF_(reduce h)_: 3.551, 3.552V, 4.0 eV → WF_(2[CaCl]+H:e−)_: 4.07 eV).

**Table 1:** Work function of inorganic electrides.

Electrides	WF	Electrides	WF	Electrides	WF
2[CaCl]^+^:2e^−^	3.43 eV	Li_3_O:e^−^^[^ ^32^ ^]^	2.50 eV	Li_3_O:e^−^@BNNT^[^ ^32^ ^]^	2.10 eV
Y_2_C:2e^−^^[^ ^33^ ^]^	2.90 eV	Y_5_Si_3_:e^−^^[^ ^34^ ^]^	2.90 eV	Ca_5_Pb_3_^[^ ^35^ ^]^	2.60 eV
C12A7:e^−^^[^ ^36^ ^]^	2.40 eV	LaFeSi^[^ ^37^ ^]^	≈2.1 eV	LaRuSi^[^ ^38^ ^]^	2.39 eV

In addition, when transition metals (such as Ru) are loaded with the electrides, they have also been proven to be high‐performance promoters for ammonia synthesis (NH_3_). The low WF endows the electride with strong electron donating ability, which can provide a range of electrons to occupy the π‐orbital of N_2_, resulting in lower apparent activation energy during nitrogen cracking process. In a recent work, Kuganathan et al.^[^ ^54^ ^]^ found that the strong electron‐donating feature of C12A7:e^−^, the strong trapping ability of Ru clusters on N_2_, the polarization of Ru_2_ clusters, and the electrostatic interaction between negatively charged N species and surface cations promote the dissociation of N_2_. Inspired by this work, we load a layer of Ru atoms on 2[CaCl]^+^:2e^−^, as shown in Figure 4b. One can find that the apparent activation energy barriers of the initial and final states relative to the intermediate states are 1.59 and 2.34 eV, respectively, which are lower than those of pure Ru metal.^[^ ^33^ ^]^ The detailed processes of N_2_ fixation in Ru/2[CaCl]^+^:2e^−^ are shown in Figure S3–S5 and Table SI and SII, Supporting Information. More recently, topological materials are widely utilized in various catalytic fields.^[^ ^55^, ^56^, ^57^, ^58^ ^]^ By applying a slight strain to break the Dirac state, we investigated the impact of 2[CaCl]^+^:2e^−^/Ru on N_2_ adsorption and dissociation. The results indicate that breaking the Dirac state does not significantly change the dissociation barrier of N_2_. Furthermore, we also study the effect on N_2_ dissociation when the Ru6 cluster is loaded with 2[CaCl]^+^:2e^−^. The results indicate that Ru6 cluster is favorable to the adsorption of N_2_, but unfavorable to the dissociation of N_2_. Compared to Ru layer, the formation of Ru6 cluster increases the potential barrier of N_2_ dissociation into two N atoms by 0.92 eV, as shown in Figure S6, Supporting Information.

## 4 Conclusion

In conclusion, based on the electron localization function, charge difference density, partial charge density, and density of states, we revealed that 2[CaCl]^+^:2e^−^ belongs to a typical 0D inorganic electride. The interstitial electrons form a tetragonal lattice under the constraint of the atomic layers. Furthermore, interstitial electrons form a highly anisotropic Dirac fermion at the X and M points, which is protected by nonsymmorphic symmetries. Remarkably, 2[CaCl]^+^:2e^−^ exhibits a lower WF (Φ_WF_  = 3.43 eV), which endows the electride 2[CaCl]^+^:2e^−^ with strong electron‐donating ability. This feature greatly promotes the cracking barrier of nitrogen on the surface of Ru/2[CaCl]^+^:2e^−^. We found that the splitting of *N_2_ into two *N atoms on the surface of Ru/2[CaCl]^+^:2e^−^ requires overcoming a potential barrier of 1.59 eV.

## Conflict of Interest

The authors declare no conflict of interest.

## Supporting information

Supplementary Material

## Data Availability

The data that support the findings of this study are available from the corresponding author upon reasonable request.
