# Supplementary material for: Zero‐Dimensional Interstitial Electron‐Induced Spin–Orbit Coupling Dirac States in Sandwich Electride
Source: Small Sci. 2024 Jul 5;4(9):2400131. doi: 10.1002/smsc.202400131 (PMC11935133; doi:10.1002/smsc.202400131)
Supplement: Supplementary file 1 — Supplementary Material [file SMSC-4-2400131-s001.pdf]

Zero-dimensional interstitial electrons induced spin-orbit coupling Dirac states in sandwich electride

Weizhen Meng[1], Jiayu Jiang[2], Yalong Jiao[1], Fengxian Ma[1], Ying Yang[3], Zhenxiang Cheng[4*], Xiaotian Wang[4*]

[1] College of Physics, Hebei Key Laboratory of Photophysics Research and Application, Hebei Normal University, Shijiazhuang, 050024, China.

[2] State Key Laboratory of Reliability and Intelligence of Electrical Equipment, and School of Materials Science and Engineering, Hebei University of Technology, Tianjin, 300130, China.

[3] College of Physics and Electronic Engineering, Chongqing Normal University, Chongqing 401331, China

[4] Institute for Superconducting and Electronic Materials (ISEM), Faculty of Engineering and Information Sciences, University of Wollongong, Wollongong, New South Wales 2500, Australia.

[*]Email: cheng@uow.edu.au; xiaotianw@uow.edu.au

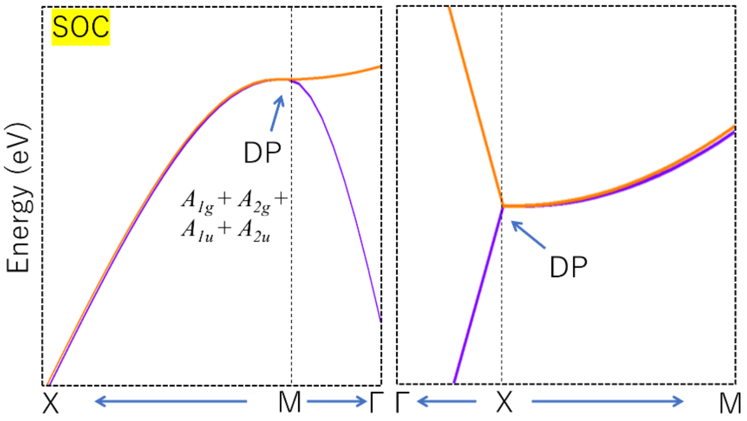

Fig. S1 Localized amplified electronic band structure at X and M points under SOC.

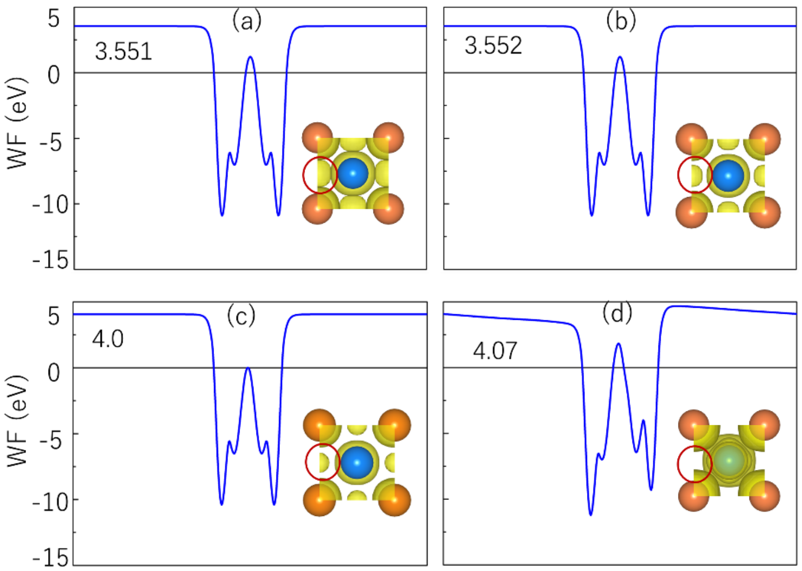

Fig. S2 (a) – (c) Work function of $2[CaCl]^{+}:2e^{-}$ by reducing interlayer spacing (h). (d) Work function of $2[CaCl]^{+}H:e^{-}$.

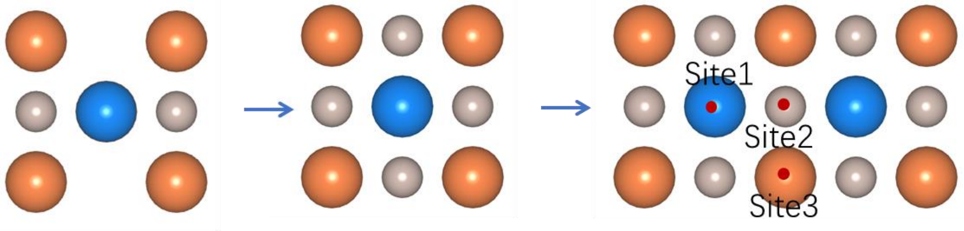

Fig. S3 Optimal crystal structures of $2[CaCl]^{+}:2e^{-}/Ru$ and the corresponding three sites (adsorbed N_2).

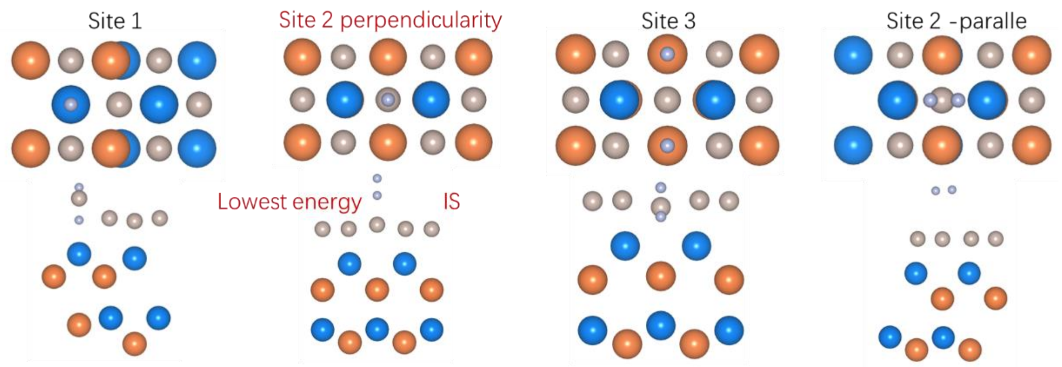

Fig. S4 Optimal crystal structures of 2[CaCl]$^+$:2e$^-$/Ru/*N_2. Table SI indicates that Site 2 is the best adsorption site.

Table SI Adsorption energy of N_2 at three sites.

Site 1 (Adsorption energy)	-2.98	Site 3 (Adsorption energy)	-2.59
Site 2 (Adsorption energy)	-3.39	Site 2 (Adsorption energy) paralle	-3.01

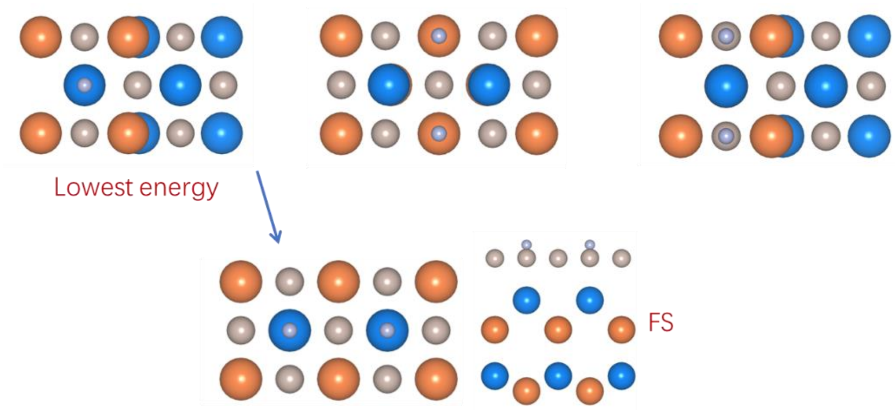

Fig. S5 Optimal crystal structures of 2[CaCl]$^+$:2e$^-$/Ru/*N. Table SII indicates that Site 1 is the best adsorption site.

Table SII Adsorption energy of N at three sites.

Site 1 (Adsorption energy)	-1.29	Site 1 (Adsorption energy)	-1.15
Site 2 (Adsorption energy)	-0.73		

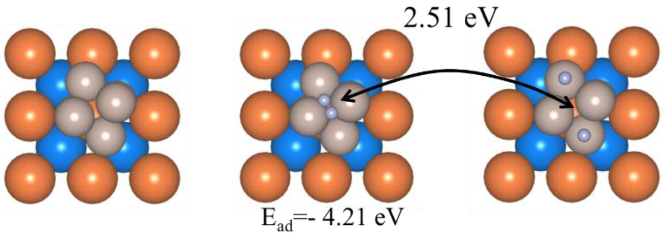

Fig. S6. Energy profiles for N_2 activation on Ru6 cluster@2$[CaCl]^+$:$2e^-$.
